# Inhibition of miR-378a-3p by Inflammation Enhances IL-33 Levels: A Novel Mechanism of Alarmin Modulation in Ulcerative Colitis

**DOI:** 10.3389/fimmu.2019.02449

**Published:** 2019-11-20

**Authors:** Karen Dubois-Camacho, David Diaz-Jimenez, Marjorie De la Fuente, Rodrigo Quera, Daniela Simian, Maripaz Martínez, Glauben Landskron, Mauricio Olivares-Morales, John A. Cidlowski, Xiaojiang Xu, Guangping Gao, Jun Xie, Jonás Chnaiderman, Ricardo Soto-Rifo, María-Julieta González, Andrea Calixto, Marcela A. Hermoso

**Affiliations:** ^1^Innate Immunity Laboratory, Immunology Program, Faculty of Medicine, Biomedical Sciences Institute, Universidad de Chile, Santiago, Chile; ^2^Laboratory of Signal Transduction, Department of Health and Human Services, National Institute of Environmental Health Sciences, National Institute of Health, Durham, NC, United States; ^3^Research Sub-direction, Academic Direction, Clínica Las Condes, Santiago, Chile; ^4^Inflammatory Bowel Disease Program, Gastroenterology Department, Clínica Las Condes, Santiago, Chile; ^5^Laboratory of Integrative Bioinformatics, Department of Health and Human Services, National Institute of Environmental Health Sciences, National Institutes of Health, Durham, NC, United States; ^6^Gene Therapy Center, University of Massachusetts Medical School, Worcester, MA, United States; ^7^Molecular and Cellular Virology Laboratory, Virology Program, Institute of Biomedical Sciences, Faculty of Medicine, Universidad de Chile, Santiago, Chile; ^8^Cell and Molecular Biology Program, Institute of Biomedical Sciences, Faculty of Medicine, Universidad de Chile, Santiago, Chile; ^9^Center for Genomics and Bioinformatics, Faculty of Sciences, Universidad Mayor, Santiago, Chile; ^10^Interdisciplinary Center of Neuroscience of Valparaíso (CINV), Faculty of Sciences, Universidad de Valparaíso, Valparaíso, Chile

**Keywords:** IL-33, miR-378a-3p, ulcerative colitis, IBD, microRNA, alarmin, PPARGC1B, microRNA family

## Abstract

Ulcerative colitis (UC) is an inflammatory bowel disease (IBD) characterized by mucosa damage associated with an uncontrolled inflammatory response. This immunological impairment leads to altered inflammatory mediators such as IL-33, which is shown to increase in the mucosa of active UC (aUC) patients. MicroRNAs present a distorted feature in inflamed colonic mucosa and are potential IL-33 regulating candidates in UC. Therefore, we studied the microRNA and mRNA profiles in inflamed colonic samples of UC patients, evaluating the effect of a microRNA (selected by *in silico* analysis and its expression in UC patients), on IL-33 under inflammatory conditions. We found that inflamed mucosa (*n* = 8) showed increased expression of 40 microRNAs and 2,120 mRNAs, while 49 microRNAs and 1,734 mRNAs were decreased, as determined by microarrays. In particular, IL-33 mRNA showed a 3.8-fold increase and eight members of a microRNA family (miR-378), which targets IL-33 mRNA in the 3′UTR, were decreased (−3.9 to −3.0 times). We selected three members of the miR-378 family (miR-378a-3p, miR-422a, and miR-378c) according to background information and interaction energy analysis, for further correlation analyses with IL-33 expression through qPCR and ELISA, respectively. We determined that aUC (*n* = 24) showed high IL-33 levels, and decreased expression of miR-378a-3p and miR-422a compared to inactive UC (*n* = 10) and controls (*n* = 6). Moreover, both microRNAs were inversely correlated with IL-33 expression, while miR-378c does not show a significant difference. To evaluate the effect of TNFα on the studied microRNAs, aUC patients with anti-TNF therapy were compared to aUC receiving other treatments. The levels of miR-378a-3p and miR-378c were higher in aUC patients with anti-TNF. Based on these findings, we selected miR-378a-3p to exploring the molecular mechanism involved by *in vitro* assays, showing that over-expression of miR-378a-3p decreased the levels of an IL-33 target sequence β-gal-reporter gene in HEK293 cells. Stable miR-378a-3p over-expression/inhibition inversely modulated IL-33 content and altered viability of HT-29 cells. Additionally, in an inflammatory context, TNFα decreased miR-378a-3p levels in HT-29 cells enhancing IL-33 expression. Together, our results propose a regulatory mechanism of IL-33 expression exerted by miR-378a-3p in an inflammatory environment, contributing to the understanding of UC pathogenesis.

## Introduction

Ulcerative colitis (UC) is an inflammatory bowel disease (IBD) characterized by chronic and recurrent inflammation of the colon and rectum mucosa. UC patients are classified according to the inflammatory activity (1), and current treatment options seek to keep the patient in a state of remission or symptoms improvement (2). Inactive UC (iUC) correspond to those with normal mucosa, and active UC (aUC) present different degrees of mucosal inflammation classified using the Mayo endoscopic score (MS) (1). The multifactorial etiology of UC is associated with an abnormal immune response to commensal bacteria combined with individual's genetic factors. Hence, the following elements have been involved in the development of the altered inflammatory response in UC: dysbiosis of gut microbiota (3), the impaired intestinal epithelial barrier function (4) and a loss of tolerance to blood flow agents (5). These factors lead to the recruitment of inflammatory cells (6) causing persistent tissue damage. The UC features are associated with the increment of immunological molecules such as TNFα, IL-13, and damage-associated molecular patterns (DAMPs), or alarmins like Interleukin (IL) 33 (7–9). The inflammatory mediator IL-33 is a member of the IL-1 superfamily, interacting with the ST2L receptor and triggering a signaling pathway which induces Th2-type cytokines (10). Increased IL-33 levels in inflamed intestinal mucosa of aUC patients (11), released mainly from the intestinal colonic epithelium (12), have been proposed as an inflammation disease marker and contributing to disease severity (13).

Additionally, IL-33 induces an anti-inflammatory response mediated by regulatory T cells (Tregs) and type 2 innate lymphoid cells (ILC2) (14, 15), by recruiting and activating M2 macrophages (16). Functionally, this cytokine could contribute to inflammation resolution by releasing IL-10 from ILC2, initiating the restoration of the epithelial barrier. Importantly, IL-33 is associated with healing processes by enhancing intestinal epithelial cells (IEC) proliferation *in vitro*, and colonic mucosal restoration in a (DSS)-induced mice model of colitis (16, 17). These dual roles make IL-33 an essential molecule in IBD pathology, being especially relevant to the study of molecular mechanisms underlying its expression, and its link to the inflammation stage. Although nuclear transcription factors might control IL-33 in an inflammatory context, other regulators such as microRNAs could adjust its temporal expression at the post-transcriptional level.

MicroRNAs are small non-coding RNAs (~22 nt) that interact through their “seed sequence” with messenger RNAs (mRNAs), promoting mRNA degradation or blocking translation (18, 19). Post-transcriptional regulation by microRNAs can coordinate temporally gene expression in diverse cell types (20), impacting on several biological processes such as proliferation, differentiation, and tissue homeostasis (21). In addition, microRNAs have been associated with the pathogenesis of intestinal diseases such as colon cancer (22), and are deregulated in the inflammatory environment of intestinal mucosa in UC patients (23–28). The effects of microRNAs on immunological factors have been described before (23, 29, 30), however, there is no evidence that they have an effect on IL-33 in the UC context.

In this study, we performed gene expression microarray analysis for mRNAs and microRNAs in UC patients to explore their molecular interaction and association. A wide variety of microRNAs showed altered expression, including a microRNA family (miR-378) with an IL-33−3′UTR binding site, as demonstrated by *in silico* analysis. We hypothesize that some of these microRNAs family members have an inhibitory effect on IL-33 expression in intestinal mucosa. To demonstrate this, we analyzed biopsies from UC patients and healthy controls and produced correlation studies. A microRNA (miR-378a-3p) selected from this first analysis, was evaluated for its role on IL-33 expression through gain and loss of function *in vitro* experiments. Lastly, we investigated the microRNA's effect on IL-33 in intestinal epithelial cell line (HT-29) under an inflammatory condition. Our results support that IL-33 is regulated by microRNAs from the miR-378 family, and offer a novel mechanism to be used in exploring new therapeutic interventions in UC patients.

## Materials and Methods

### Human Samples

Colonoscopic pinch biopsies of inflamed and non-inflamed mucosa from eight active UC patients were used for microarray assays (Table 1, left). Colonic biopsies of 24 active UC patients (Mayo score 2 and 3), 10 inactive UC (Mayo score 0 and 1), and six healthy subjects for qPCR assays were added (Table 1, center). From the active UC patient cohort, 16 colonoscopic samples were included for the evaluation of IL-33 protein levels by ELISA (Table 1, right). Recruitment was conducted by the gastroenterology service of Clínica Las Condes.

**Table 1:** Clinical characteristics of patients.

	**Microarrays assays**		**qPCR assays**					**ELISA assay**	
	**Active UC**		**Inactive UC**		**Active UC**		**Controls**	**Active UC**	
No. of patients	8		10		24		6	16	
Mayo score	MS = 2	MS = 3	MS = 0	MS = 1	MS = 2	MS = 3		MS = 2	MS = 3
	7	1	4	6	14	10		7	9
Female, *n* (%)	5 (62.5%)		5 (50%)		16 (66%)		5 (42%)	8 (50%)	
Age (y)									
Median	34		46		35		52.5	31.5	
Range	18–44		20–76		24–60		36–70	24–53	
Duration of IBD (y)									
Median	3		10		4		–	5.5	
Range	1–7		0.6–21		0.2–21			0.2–21	
Treatments									
5 ASA	2		3		5		0	5	
Steroids	1		0		3		0	3	
Steroids + 5 ASA	1		0		2		0	2	
Steroids + Immunosuppressant	2		0		4		0	4	
Steroids + Sulfasalazine	0		1		2		0	2	
Sulfasalazine	0		1		0		0	0	
5 ASA + immunosuppressant	1		3		0		0	0	
Methotrexate + 5 ASA	0		1		0		0	0	
Biologics therapy									
Biologics therapy + 5 ASA + Sulfasalazine	0		1		3		0	0	
Biologics therapy + Immunosuppressant	1		0		3		0	0	
Biologics therapy + 5 ASA	0		0		1		0	0	
Biologics therapy + immunosuppressant + 5 ASA	0		0		1		0	0	

### Ethics Statement

All participants provided informed consent. The study was approved by the Sub-direction of Research, and Local Ethics Committee from Clínica Las Condes (Act accepted the August 8, 2014) and performed according to human experimental guidelines. Clinical investigation was conducted according to Declaration of Helsinki principles with participants identified only by number.

### Total RNA and microRNA Enrichment

Total RNA for microarrays was obtained using Mirvana microRNA Kit (Life Technologies Carlsbad, CA), and RNA integrity was evaluated with the High Sensitivity RNA Analysis Kit (Advanced Analytical Technologies Ankeny, IA). For qPCR analysis, large mRNAs (>200 nt) from the colonic biopsies of patients were isolated using miRNeasy Kit (Qiagen Germantown, MD), and an enriched fraction of small RNAs was obtained with the Rneasy mini elute clean up kit (Qiagen Germantown, MD).

### Microarray Assays

MicroRNA and gene expression microarrays of inflamed and non-inflamed mucosa from eight active UC patients was done by using the Affymetrix GeneChipTM Version 4 microRNA arrays following the Affymetrix hybridization protocols (ThermoFisher Scientific Santa Clara, CA). This platform has extended coverage of more than 5,000 mature microRNAs (included in the miRBase 20 release). Starting with 350 ng of total RNA, the Affymetrix FlashTagTMBiotin HSR labeling was used to label RNA according to the manufacturer's protocol. On average 13 μg of labeled sample were hybridized for 16 h at 48°C using the Affymetrix Eukaryotic Target Hybridization Controls protocol. Array slides were stained with streptavidin/phycoerythrin utilizing a double-antibody staining procedure and then washed for antibody amplification according to the GeneChip Hybridization, Wash, and Stain Kit was used according to the manual. Arrays were scanned in an Affymetrix Scanner 3000 and data was obtained using the GeneChip® Command Console Software (AGCC; Version 3.2). Gene expression analysis was conducted using Agilent Whole Human Genome 4×44 multiplex format oligo arrays (014850) (Agilent Technologies Santa Clara, CA) that has coverage of more than 40,000 human mRNA transcripts. Fifty nanograms (ng) of total RNA were amplified as directed in the NuGEN Ovation Pico WTA System and 5 μg of amplified cDNA were enzymatically labeled using the Agilent SureTag DNA Labeling Kit. For each sample, 3 μg of Cy3 labeled cDNA were hybridized for 17 h. Slides were washed and then scanned with an Agilent Scanner. Data were obtained using the Agilent Feature Extraction software (v12), using the 1-color defaults for all parameters. The Agilent Feature Extraction Software performed error modeling, adjusting for additive and multiplicative noise. The resulting data of microRNA and gene expression microarrays were processed using OmicSoft Array Studio (Version 9.0) software. Raw data were processed with Partek® Genomic Suite® software, version 6.6 Copyright, 2016 Partek Inc., to generate a Principal Components Analysis and to find gene probes expressed with statistical significance. Ingenuity® Pathway Analysis (IPA®) version 6.5 tool (QIAGEN Inc.) Ingenuity Systems 2 was used to identify functional pathways and top upstream regulators along with their statistical significance. The network relation between microRNA and transcripts figure was generated through the use of IPA®. Gene expression and small RNAs microarrays are linked to GEO accession GSE133061.

### RNA-RNA Interaction Analysis

The prediction of interaction between RNA molecules was using the INTA RNA 2.1.0 and Vienna RNA package 2.1.3 (31). Minimum Free Energy (MFE) analysis to predict the probability of 3′UTR RNA folding was conducted using the Vienna RNA tool (32). Identification of target sites for microRNA was conducted using TargetScan 7.2 (33).

### Quantitative Real-Time PCR

Taqman MicroRNA Assay (ThermoFisher Scientific Carlsbad, CA) was used to analyze the expression of miR-378a-3p, miR-378c, and miR-422a and the endogenous control RNU43 at full reaction volume (20 μl) according to the manufacturer's instructions. To evaluate the levels of each mRNA, the total cDNA was reverse transcribed from 1 μg of RNA using 1 unit of reverse transcriptase (Agilent Technologies). Quantitative real-time PCR for IL-33, IL-8 AGO1, AGO2, PPARGC1B, and 18s rRNA genes were performed using II Brilliant SybrGreen qPCR Master Mix (Agilent Technologies). Primer sequence for IL-33 Fwd GTGACGGTGTTGATGGTAAGATGT, Rev CACTCCAGGATCAGTCTTGCAT; PPARGC1B Fwd TGTTCAGACAGAACGCCAAG, Rev AAGCCGTACTTCTCGCCTCT; IL-8 Fwd TCTGGACCCCAAGGAAAACT, Rev TTGCATCTGGCAACCCTACA; AGO1 Fwd GGTCCAGCATTTCAAGCCTCAGAT, Rev CACAATGGCTAGCCACTTGATGGA; AGO2 Fwd GGGGCAGGAATAAAGCTATTGCGA, Rev TGCGCGTATTTGCAGAAGCAC; 18s ribosomal RNA (rRNA) Fwd GTGGAGCGATTTGTCTGGTT, Rev CGCTGAGCCAGTCAGTGTAG. The 18s rRNA was used as a reference gene to normalize mRNA levels. Each sample was measured by duplicated with qPCR results analyzed by the ΔΔC_q_ method.

### Western Blotting

Proteins extracted using RIPA Lysis Buffer (ThermoFisher Scientific Carlsbad, CA) supplemented with Complete mini protease inhibitors cocktail (Roche, Mannheim, Germany) and immersed in Laemmli Buffer (Sigma Aldrich Saint Louis, MO) from HT-29 experiments (HT-29: 40 μg, HT-29 transduced cell lines: 30 μg) were loaded in 10% polyacrylamide gels. Protein transference to nitrocellulose membranes was accomplished using a Trans-Blot Turbo Transfer System (Bio Rad Laboratories Inc). Membranes were incubated overnight with Monoclonal IL-33 antibody MAB3625 (R&D Minneapolis, MN) (1:250) and β-actin as load control (1:2,000) (Santa Cruz Biotechnology Dallas, TX). Chemiluminescence was evaluated using ChemistCope equipment and the bands area were quantified with the Image J Software.

### ELISA

Protein extracts (100 μl) with RIPA Lysis Buffer (ThermoFisher Scientific Carlsbad, CA) supplemented with protease inhibitors cocktail (Roche, Mannheim, Germany) were used to measure IL-33 concentration from human tissue samples by ELISA (DuoSet, R&D Systems, Minneapolis, MN, USA) according to the manufacturer's instructions. Each sample was duplicated and average results were normalized to total protein concentration and expressed as pg (IL-33)/mg (total protein).

### Cell Lines and Cultures

HT-29 was kindly provided by Dr. Julio Tapia (Cell Transformation Laboratory, Faculty of Medicine, University of Chile). HT-29 and HEK 293 cells were cultured in high glucose DMEM (Corning Inc., Tewksbury, MA) with 10% Fetal Bovine Serum (FBS, Hyclone Pittsburgh, PA), Penicillin/Streptomycin (1%). HEK 293T cells were cultured in MEM medium (Corning Inc., Tewksbury, MA) 10% SFB and Penicillin/Streptomycin (1%).

### Lentivirus Preparation

For stable over-expression of miR-378a-3p in HT-29 cells, we used the primiR-378 sequence (300 pb flanking the miR-378a stemloop:MI0000786) inserted in the SalI site of vector pGPG-Lenti-CMV-GFP-puro vector previously validated for microRNA expression (34). The cloned vector, pGPG-Lenti-CMV-PrimiR-378 plasmid, or negative vector pGPG-Lenti-CMV-GFP-puro were co-transfected with the helper plasmids (pPAX2 and pVSV-G) in HEK 293T cells. Supernatants with lentiviral particles were recovered 48 h post-transfection and the cell debris removed by centrifugation. Stable miR-378a-3p inhibition was achieved using Tud Decoy technology. LentiMiR378a-3pTuD and negative scrambled control #1 were purchased (MISSION® Lenti microRNA Inhibitors, Sigma Aldrich, St. Louis, MO). PrimiR-378, control primir-378, MiR-378a-3p (TuD) Inhibitor or TuD Inhibitor control lentiviral particles were added to HT-29 cells previously treated with Polybrene 6 μg/mL (Sigma-Aldrich St. Louis, MO) and incubated for 72 h at 37°C with 5% CO_2_ environment on 24-well culture plates. Then, Puromycin (2 μg/mL) selection was performed for 2 weeks.

### Gen Reporter Assays

The IL-33 3′UTR wild type binding sequence (42 nt) was cloned into a β-galactosidase pmiRCHECK reporter vector into the PmeI restriction site (IL-33 WT). A control plasmid with interspersed substitutions in the seed sequence was cloned into the same vector (IL-33 MUT). A β-gal vector containing 3 canonic binding sites sequence for miR-378a-3p was used as a positive control (pmirCHECK 3′UTR XXX MiR-378a-3p BS). MicroRNA sensor vectors and positive control are tools previously validated in the Gene Therapy Center (UMASS) and published for microRNA effect evaluation in HEK 293 cells (34, 35). HEK293 cells (2.5 × 10^4^ cells) were seed in 24 wells plates in 500 ul of DMEM medium with SFB (10%). After 24 h the transfection with Mimics Negative control #1 (ThermoFisher Scientific Carlsbad, CA) 10 nM, Mimic 5 nM plus Mimic negative control 5 nM, and Mimics 10 nM, and co-transfected with 200 ng of β-gal vector using Lipofectamine 2000 (ThermoFisher Scientific Carlsbad, CA) according to manufacturer instructions were accomplished. β-Gal levels (Applied Biosystems, Bedford, MA, USA) were measured 24 h post-transfection and normalized to Luciferase expression (F-Luc) (Promega Madison, WI) in four duplicate independent experiments according to manufacturer instructions.

### Cell Viability Assay

HT-29 over-expressing or inhibiting of the miR-378a-3p and their respective cell line controls were seeded (3 × 10^5^ cells) in 6-wells plates with DMEM, FBS (10%) and Penicillin/Streptomycin (1%). Afterward, cells were trypsinized at 6, 12, 24, 48, and 72 h and the number of viable cells (unstained) were counted by trypan blue stain (Thermofisher Scientific Carlsbad, CA) using a Neubauer Camera. Additionally, viable cells were determined by flow cytometry (FACSCanto Becton Dickinson Biosciences, San Jose CA, USA) using propidium iodide (PI) stain (10 μg/mL) (ThermoFisher Carlsbad, CA, USA) at 24, 48, and 72 h in four duplicate independent experiments.

### Inflammatory Assay in Intestinal Epithelial Cells

HT-29 1 × 10^6^ cells were incubated in DMEM with 1% FBS overnight and then treated with human recombinant TNFα (R&D Systems, Minneapolis, MN USA) at 10 ng/mL for 3, 6, and 24 h for microRNA and mRNA evaluation, and 12, 24, and 48 h for protein measurement. Four duplicate independent experiments were performed.

### Statistical Analysis

Microarrays results were analyzed using two-way ANOVA to determine significant differences between genes and microRNAs probes. A cutoff of 1.5-fold change, a *P* ≤ 0.05 (microRNAs) and ≤ 0.01 (mRNAs) were used to select the gene probes for further analysis. Statistical analysis for qPCR, ELISA, chemiluminescence and luminescence assays were performed with ANOVA and 2-tailed Student test (GraphPad 7.0) unpaired or paired according to sample characteristics. Results are expressed as Mean values ± standard error (SEM). Bonferroni and Tukey post-tests were used for ANOVA analysis. For correlation analysis, we used Pearson correlation. *P* ≤ 0.05 was considered significant.

## Results

### Expression of microRNAs and mRNAs Associated With Inflammatory and Metabolic Pathways Is Altered in Inflamed Tissue

**Figure 1:**
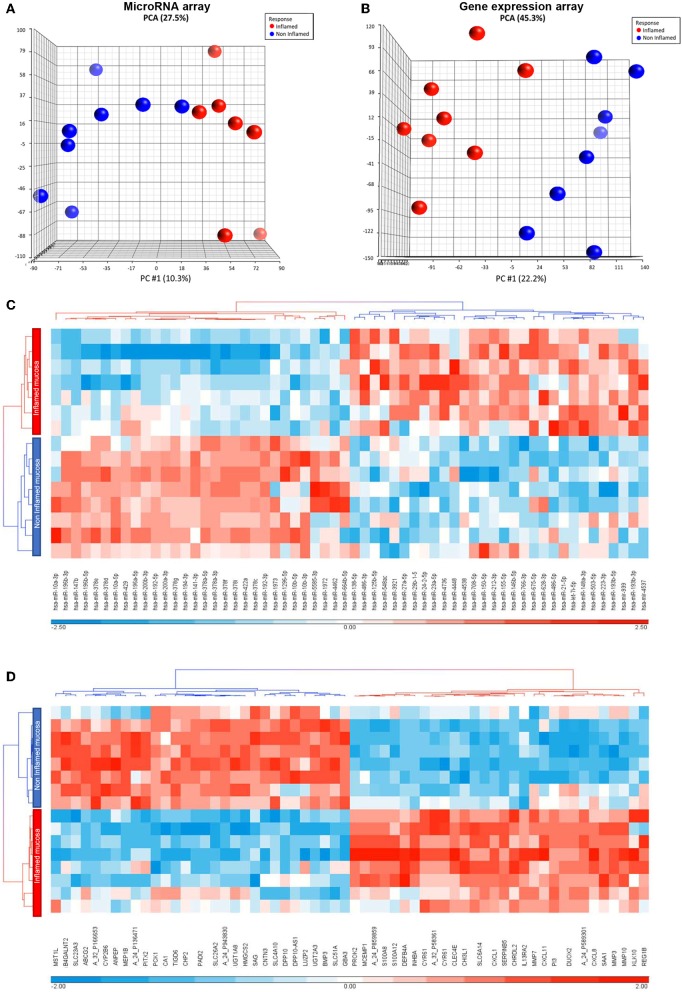
Inflammation context impact on microarrays analysis. **(A)** Total principal component analysis (PCA) and the main component PC #1, corresponding to variation by inflammation, performed in inflamed and non-inflamed colon biopsies from aUC patients for microRNA microarray and **(B)** gene expression microarray. **(C)** Cluster analysis for microRNA microarray and **(D)** gene expression microarray. Hierarchical clustering was performed based on the main 30 up and downregulated selected gene probes.

To have an overview of transcripts and microRNAs involved in inflammatory pathways, particularly related to increased IL-33 levels in aUC (11), we determined their differential expression in inflamed vs. non-inflamed intestinal mucosa (from colon pinch biopsies), by microarray assays. Clinical characteristics of each patient are described in Table 1. To provide an overview of variance in samples given by the inflammatory mucosa context, we performed a Principal Component Analysis (PCA) of microRNAs and mRNAs arrays. This analysis indicates that inflammation condition impact on molecules expression as seen in the main component (PC #1), explaining 10.3 and 22.2% of total variances (27.5; 45.3%), respectively, for the microRNAs and mRNAs arrays (Figures 1A,B, respectively). To visualize the impact of inflammation on microRNA and mRNA expression, we performed a Heat map analysis (Figures 1C,D, respectively). A total of 89 mature microRNAs and 3,854 gene expression probes were regulated by the inflammatory context (≥1.5-fold). For microRNA, there were a similar number of up-regulated (36) and down-regulated (37) probes modulated by inflammation condition. Additionally, there were slightly more up-regulated transcripts (2,120) than those down-regulated (1,734) by inflammation in gene expression microarray.

To understand inflammation impact on differentially expressed miRNA and transcript expression and signaling pathways, we used the Ingenuity Pathway Analysis (IPA) software. Mitochondrial dysfunction, adhesion of granulocytes and diapedesis were the most commonly affected pathways by inflammation (Table 2). The tumor necrosis factor (TNF) was the upstream regulator molecule mostly related to altered mRNAs expressed in inflamed mucosa. Additionally, differentially expressed genes were associated with cancer, gastrointestinal, immunological diseases, and cellular processes such as cell movement, growth, and proliferation (Table 2).

**Table 2:** Analysis of functional groups from gene expression microarray by IPA.

	**Top canonical pathways**	**Top upstream regulators**	**Top molecular and cellular functions**
1.	Mitochondrial dysfunction	TNF	Cellular movement
2.	Granulocyte adhesion and diapedesis	LPS	Cellular growth and proliferation
3.	Oxidative phosphorylation	β-estradiol	Cellular development
4.	Antigen presentation pathway	TGFβ1	Cell-to-cell signaling and interaction
5.	Role of tissue factor in cancer	IFNγ	Cell Death and survival

Subsequently, we analyzed strongly deregulated transcript fold-change such as IL-33 to evaluate possible relationships with the inflammatory environment. We observed large up-regulation of MMP3 (59.5 times), CXCL8 (40.1 times) (Table 3), along with a high IL-33 mRNA fold-change (3.8 times). Alternatively, most down-regulated genes were HMGCS2 (−19 times) and SCL26A2 (−14.5 times). Regarding microRNA expression, we found a robust up-regulation of miRNAs related to immune homeostasis such as miR-hsa-miR-223-3p and miR-708-5p (Table 3), suggesting that inflammation is an important feature impacting in microRNA profile. In addition, eight members of the microRNAs miR-378 family were among the most down-regulated genes (Table 3). To find predicted mRNA targets for this microRNA family, we performed an interaction network using IPA software, which uses data derived from TargetScan, TarBase miRecords and Ingenuity® Knowledge Base. This analysis shows a direct relationship between the miR-378 family and the IL-33 transcript (Supplementary Figure 1A). This microRNA family shares the same seed sequence and, thus might interact with IL-33 mRNA. TargetScan software also predicted the interaction between these molecules and identified that the type of pairing between miR-378 family seed sequence within IL-33 mRNA is through an 8mer in the 3′UTR (Supplementary Figure 1B), exhibiting an effective canonical site type (33). Furthermore, we analyzed the 3′UTR IL-33 sequence based in MFE to evaluate the folding interactions (Vienna RNA software). Accordingly, the location of target sequence in 3′UTR IL-33 has a moderate probability in presenting an unpaired conformation (Supplementary Figure 1C), suggesting that the IL-33 binding site is accessible for microRNA interaction. Additionally, to predict an interaction between the miR-378 family and IL-33 3′UTR, we evaluated the binding strength of both molecules by interaction energy analysis (IntaRNA-RNA-RNA Interaction software) (Table 4). The lowest interaction energy was found for miR-422a (−13.6 kcal/mol) and miR-378c (−9.6 kcal/mol), energies acceptable for human microRNAs interactions (38). In conjunction, these analyses suggest that miR378 family members interact with IL-33 3′UTR. Based on these findings and background information involving these microRNAs and intestinal mucosa inflammation, we selected miR-378a-3p [down-regulated in inflamed mucosa (23)], miR422a and miR-378c for further correlation analysis in a cohort of patients with aUC, inactive UC (iUC) and healthy controls.

**Table 3:** Top microRNAs and mRNAs deregulated in inflamed mucosa.

**Induced microRNAs ID**	**Fold change**	**Repressed microRNAs ID**	**Fold change**	**Induced mRNAs ID**	**Fold change**	**Repressed mRNAs ID**	**Fold change**
hsa-miR-223-3p	5.3	hsa-miR-147b	−6.6	MMP3	59.5	HMGCS2	−19
hsa-miR-708-5p	4.7	hsa-miR-196b-3p	−6.4	CHRDL2	50.5	SLC26A2	−14.5
hsa-miR-29b-1-5p	3.0	^*^hsa-miR-378e	−3.9	PROK2	50.5	UGT2A3	−13.8
hsa-miR-138-5p	2.9	^*^hsa-miR-422a	−3.9	SLC6A14	45.1	PITX2	−12.5
hsa-miR-23a-5p	2.8	^*^hsa-miR-378g	−3.8	CXCL8	40.1	SLC51A	−12.4
hsa-miR-486-3p	2.8	hsa-miR-196b-5p	−3.6	MMP7	38.3	BMP3	−11.8
hsa-miR-27a-5p	2.8	^*^hsa-miR-378d	−3.5	DUOX2	35.4	B4GALNT2	−10.9
hsa-miR-766-3p	2.8	^*^hsa-miR-378a-3p	−3.4	SLC6A14	33.3	TIGD6	−10
hsa-miR-155-5p	2.5	hsa-miR-378a-5p	−3.4	PI3	32.3	DPP10	−9.9
hsa-miR-4538	2.5	hsa-miR-192-3p	−3.1	DUOX2	30.9	SAG	−9.8
hsa-miR-503-5p	2.4	^*^hsa-miR-378c	−3.1	SAA1	27.3	CA1	−9.7
hsa-miR-4448	2.3	^*^hsa-miR-378i	−3.1	CHI3L1	25.0	UGT1A8	−9.4
hsa-miR-548ac	2.3	^*^hsa-miR-378f	−3.0	CYR61	24.4	PCK1	−9.1
hsa-miR-4736	2.2	hsa-miR-141-3p	−2.8	SERPINB5	23.0	MST1L	−8.7
hsa-miR-193b-5p	2.1	hsa-miR-1296-5p	−2.7	INHBA	22.9	DPP10-AS1	−8.6

* *miR-378 family members*.

**Table 4:** Interaction energy analysis between miR-378 family and IL-33 3′UTR using INTA RNA software.

**MicroRNA**	**Energy: kcal/mol**
miR-422a	−13.61
miR-378c	−9.61
miR-378i	−9.46
miR-378f	−8.67
miR-378d	−8.62
miR-378a-3p	−8.47
miR-378g	−8.34
miR-378e	−7.14

### MiR-378a-3p Correlates Inversely With IL-33 Expression in Intestinal Mucosa of UC Patients and Is Modulated by Anti-TNF Therapy

**Figure 2:**
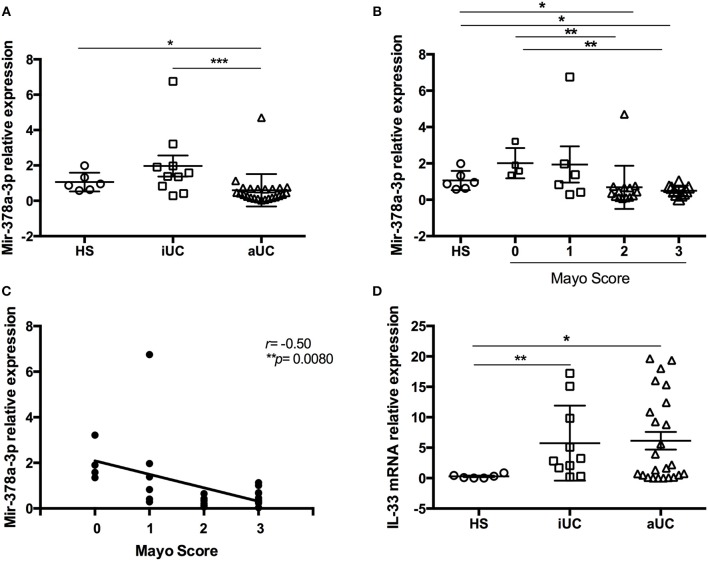
MiR-378a-3p levels are decreased in intestinal mucosa from active UC patients. **(A)** Relative expression from healthy subjects (HS) (6), inactive (iUC) (10), and aUC (24) patients of miR-378a-3p by qPCR, **(B)** miR-378a-3p levels according Mayo Score (MS), and **(C)** correlation analysis between miR-378a-3p and MS, and **(D)** IL-33 mRNA by qPCR. Mann Whitney test and Pearson Correlation were performed. *P = < *0.05*, **P = < *0.01*, ***P = < *0.01*.

**Figure 3:**
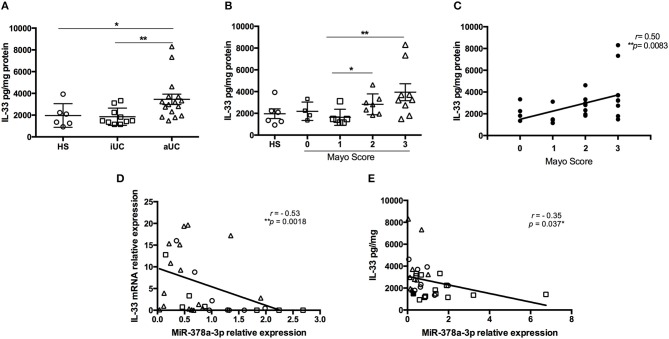
MiR-378a-3p levels are inversely correlated with IL-33 content in intestinal mucosa from UC patients and healthy subjects. **(A)** IL-33 protein levels by ELISA of HS = 6, iUC = 10 and aUC = 16, **(B)** according to MS, and **(C)** correlation analysis between IL-33 and MS. **(D)** Correlation analysis between miR-378a-3p and IL-33 mRNA, and **(E)** with IL-33 protein. Mann Whitney test and Pearson Correlation were performed. *P = < *0.05*, **P = < *0.01*.

To corroborate levels of miR-378a-3p, miR-378c, miR422a, and IL-33 observed by microarrays, we enrolled aUC and inactive UC (iUC) patients together with a healthy subject group for their evaluation by qPCR. Analysis showed that aUC patients had decreased miR-378a-3p levels compared to healthy subjects (HS) (*P* = 0.018) and with iUC (*P* = 0.0006) (Figure 2A). When we evaluated the miR-378a-3p levels according to MS, we found lower levels of this microRNA mainly in patients with MS = 2, 3 compared to MS = 0 (*P* = 0.0078, *P* = 0.002, respectively) (Figure 2B), and a negative correlation with the MS (*r* = −0.50, *P* = 0.0080) (Figure 2C). IL-33 mRNA was found increased in aUC and iUC patients compared to HS (*P* = 0.023, *P* = 0.0075, respectively) (Figure 2D). Also, protein IL-33 levels were increased in intestinal mucosa lysate of aUC vs. HS or iUC (*P* = 0.046, *P* = 0.0034, respectively) (Figure 3A). Analysis showed the highest IL-33 protein content was in patients with MS = 2 and MS = 3 compared to MS = 1 (*P* = 0.022, *P* = 0.0048, respectively) (Figure 3B) along with direct correlation between them (*r* = 0.50, *P* = 0.0083) (Figure 3C). Moreover, miR-378a-3p and IL-33 (mRNA and protein) levels were found inversely correlated (*r* = −0.53, *P* = 0.0018; *r* = −0.35, *P* = 0.037, respectively) (Figures 3D,E).

Regarding other microRNA expression in colonic biopsies, we determined a reduction of miR422a levels in aUC compared to iUC patients (*P* = 0.0077) (Supplementary Figure 2A), with no differences between UC patients and HS. For miR-378c, there were no differences between HS, iUC and UC samples (Supplementary Figure 2B). Levels of miR-422a correlated inversely with IL-33 protein (*r* = −0.36, *P* = 0.037) (Supplementary Figure 3A), but not with IL-33 mRNA levels (Supplementary Figure 3B), and miR-378c were no correlated with IL-33 expression either (Supplementary Figures 3C,D). These results suggest that miR-378a-3p and miR-422a might be potential regulators of IL-33 expression.

TNFα is a pro-inflammatory molecule that is increased in inflamed UC intestinal mucosa patients (39), regulating different microRNAs (40). Thus, the possible effect of TNFα on expression of the three microRNAs included in this study was determined in inflamed mucosa of patients (MS = 2–3) receiving anti-TNFα therapy (*n* = 8) compared with aUC with other treatments (*n* = 16). We found increased levels of miR-378a-3p and miR-378c (*P* = 0.036, *P* = 0.01, respectively) (Supplementary Figures 4A,B, respectively), along with decreased IL-33 mRNA levels (*P* = 0.031) (Supplementary Figure 4C) in inflamed mucosa of patients with anti-TNFα therapy. MiR-422a levels in mucosa patients with this treatment were not altered (Supplementary Figure 4D). These findings suggest an inhibitory effect of TNFα on miR-378a-3p and miR-378c levels.

We show that miR-378a-3p expression is inversely correlated with IL-33 mRNA and protein; IL-33 mRNA is a predicted target of miR-378a-3p and this microRNA is potentially regulated by TNFα. Given these reasons we selected miR-378a-3p to validate its effect on IL-33 through an *in vitro* approach and to further study their modulation by TNFα.

### IL-33 Target Sequence Is Inhibited by miR-378a-3p

**Figure 4:**
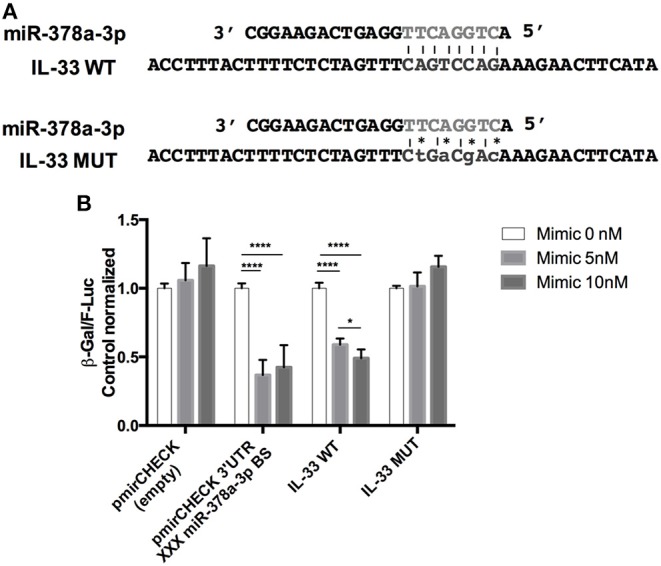
Identification of putative miR-378a-3p binding site within the IL-33 3′UTR. **(A)** Diagram of 3′UTR IL-33 target sequence into Lac Z reporter gene to evaluate microRNA effect, **(B)** miR-378a-3p over-expression reduces β-Gal activity of a reporter vector harboring IL-33 wild-type target sequence in HEK 293T cells. Mimic 0 nM (mimic negative control 10 nM), Mimic miR-378a-3p 5nM + Mimic negative control 5 nM, or Mimic 10 nM were co-transfected with sensor vectors. β-Gal F-Luc ratios of Mimic miR-378a-3p treatments were normalized to Mimic 0 nM of each sensor vectors. Four experiments by duplicated were analyzed using Two-way ANOVA with Tukey's multiple comparisons test. *P = < *0.05*, ****P = < *0.0001*.

**Figure 5:**
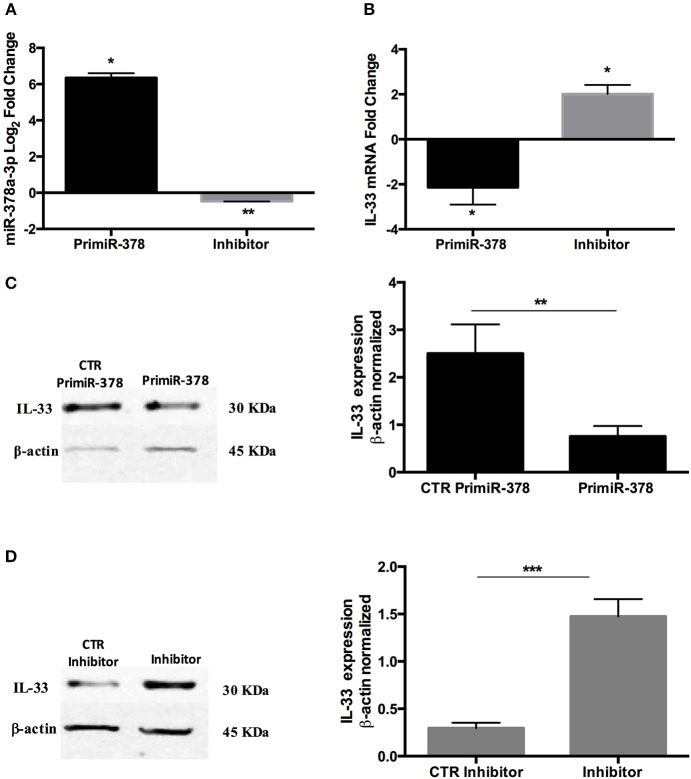
Over-expression and inhibition of miR-378a-3p modulate IL-33 expression. Stable expression of miR-378a-3p (Primir-378) or inhibition of miR-378a-3p (Inhibitor), and their respective controls (CTR PrimiR-378a, CTR Inhibitor) were obtained with Lentiviral vectors in HT-29 cells. **(A)** MiR-378a-3p Log_2_ fold change, and **(B)** IL-33 mRNA fold change relative to CTR cell lines by qPCR, **(C)** IL-33 protein and β-actin by Western Blotting (WB) and bands quantification by densitometry analysis (Fiji Software) of Control Primir-378 and Primir-378 cell lines, and **(D)** Control Inhibitor and Inhibitor cell lines. Four experiments by duplicated were performed. Each cell line was compared with their respective control using Paired *t*-test. *P = < *0.05*, **P = < *0.01*, ***P = < *0.001*.

To determine the effect of miR-378a-3p on IL-33 expression, we used a β-Galactosidase (β-Gal) gene sensor vector containing the binding site (BS) for miR-378a-3p in the wild type IL-33 3′UTR (WT IL-33), or a BS mutant vector (MUT IL-33). The annealing between the WT and MUT BS sequences with miR-378a-3p is shown in a diagram in Figure 4A. A positive control that contains 3 consecutive canonic target sequences for miR-378a-3p was used (pmirCHECK 3′UTR XXX MiR-378a-3p BS). The β-Gal activity was reduced in HEK-293 cells transfected with WT IL-33 sensor vector and Mimic 5 or 10 nM compared to Mimic 0 nM (*P* < 0.0001). This reduction was dependent on the miR-378a-3p mimic concentration used (Figure 4B) (*P* < 0.05). β-Gal activity decreased in cells transfected with the positive control as well (*P* < 0.0001), while the mimic molecule had no effect on β-Gal activity of MUT IL-33 vector. This outcome suggests that miR-378a-3p exerts an inhibitory effect on the target sequence present in the 3′UTR of IL-33. Also, we generated stable HT-29 cell lines that over-express (PrimiR-378 cells) or inhibit the miR-378a-3p (Inhibitor cells) along with their respective control cell lines (CTR Primir-378, CTR Inhibitor). The basal levels of miR-378a-3p were evaluated to determine the effectiveness of the transduction. Thus, miR-378a-3p levels increased on average 75-fold in PrimiR-378 cells and decreased 1.3-fold (30% repression) in the Inhibitor cells, relative to each control cell line (Figure 5A) (*P* = 0.011, *P* = 0.0037, respectively). The levels of IL-33 mRNA decreased 2.1-fold in HT-29 PrimiR-378 cells and was induced 2-fold in HT-29 Inhibitor cells (Figure 5B) (*P* = 0.045, *P* = 0.048, respectively. These results strongly suggest that miR-378-3p regulates IL-33 mRNA post-transcriptionally, possibly by promoting its degradation. Furthermore, IL-33 protein levels decreased in Pri-378 cells and increased in inhibitor cells compared with their respective controls (Figures 5C,D) (*P* = 0.0033, *P* = 0.0004, respectively).

**Figure 6:**
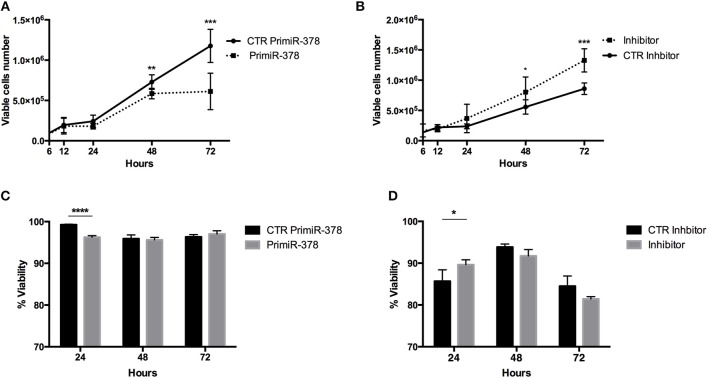
Over-expression and inhibition of miR-378a-3p levels alter viability of colonocytes cell line. **(A)** IEC cells over-expressing the primiR-378, and **(B)** inhibiting the miR-378a-3p and their respective control cell lines (CTR PrimiR-378, CTR Inhibitor) were cultured by 6, 12, 24, 48, and 72 h. The number of viable cells were counted using trypan blue stain. The % of viability was achieved with PI stain by flow cytometry in **(C)** IEC cells over-expressing the primiR-378 and **(D)** inhibiting the miR-378a-3p cultured by 24, 48, and 72 h. Four experiments by duplicated were analyzed using Two-way ANOVA with Bonferroni multiple comparisons test were performed. *P = < *0.05*, **P = < *0.01*. ***P = < *0.001*, ****P = < *0.0001*.

High IL-33 expression has been associated with increased viability (41), thus we evaluated viable PrimiR 378 and Inhibitor stable cells by the trypan blue exclusion test. Cells over-expressing miR-378a-3p (presenting low IL-33 expression) diminish the number of viable cells (*P* = < 0.01; *P* = < 0.001) (Figure 6A) compared to control cells, and conversely miR-378a-3p inhibition increased it (*P* = < 0.05; *P* = < 0.001) (Figure 6B) at 48 and 72 h. Additionally, we found decreased viability in cells over-expressing miR-378a-3p (*P* = < 0.0001) (Figure 6C), with an increment of viability in miR-378a-3p Inhibitor cells (*P* = < 0.05) (Figure 6D) at 24 h according to propidium iodide stain. In conjunction, these results show the inhibitory effect of miR-378a-3p on IL-33 expression on cell viability.

### TNFα Reduces the miR-378a-3p Levels in HT-29 Cells

**Figure 7:**
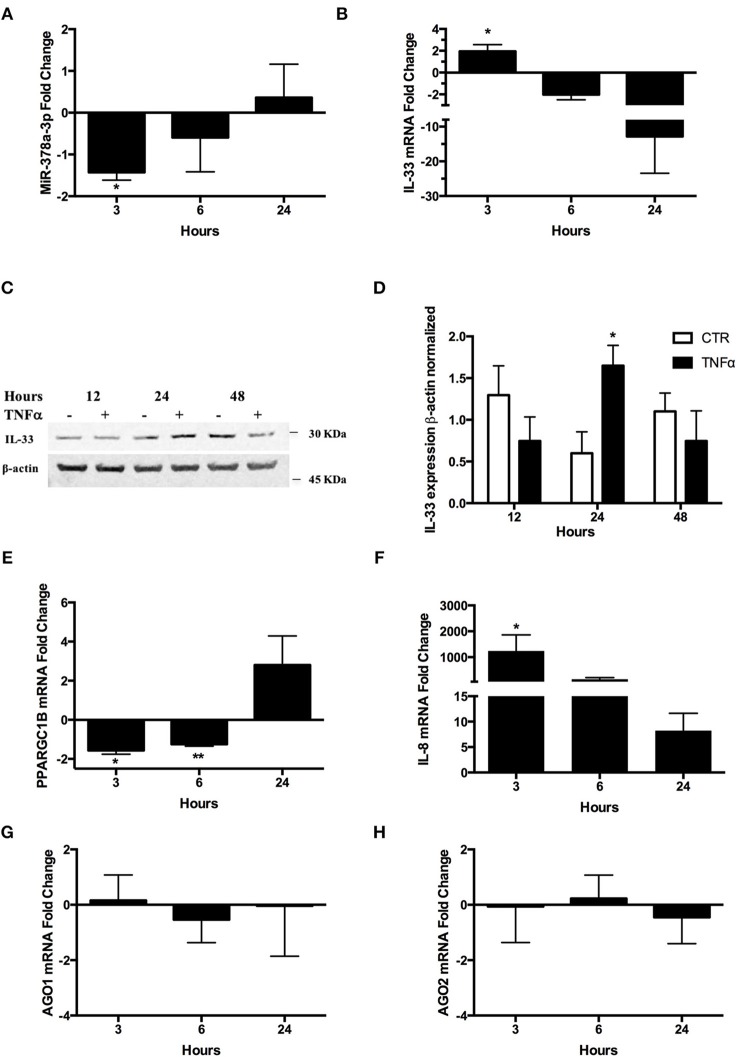
TNFα decreases miR-378a-3p and increases IL-33 expression in colonocytes. HT-29 cell line was stimulated with 10 ng/mL human recombinant TNFα (hr-TNFα) for 3, 6, and 24 h. Fold change relative to non-stimulated control of **(A)** MiR-378a-3p and **(B)** IL-33 mRNA. **(C)** IL-33 protein was measured in HT-29 cells stimulated with hr-TNFα 10 ng/mL 12, 24, and 48 h by WB, **(D)** Band quantification of IL-33 protein normalized to β-actin. **(E)** Fold change relative to non-stimulated control of PPARGC1B mRNA and **(F)** IL-8 mRNA levels (measured as control of TNFα stimulus). **(G)** AGO1 and **(H)** AGO2 mRNA levels were assessed as control of miRNA-biogenesis machinery. Four experiments by duplicated were analyzed using Two-way ANOVA with Bonferroni post-test. *P < *0.05*, **P = < *0.01*.

Due to higher miR-378a-3p levels in inflamed aUC mucosa patients receiving anti-TNFα therapy, we conducted experiments replicating the inflamed condition in a colonocyte *in vitro* model (HT-29 cells) exposed to TNFα. Levels of miR-378a-3p decreased with TNFα stimulation at 3 h (Figure 7A) whilst simultaneously IL-33 mRNA levels increased (Figure 7B) (*P* = < 0.05). Additionally, IL-33 protein increased at 24 h post-stimulus (Figures 7C,D) (*P* = < 0.05). These results showed an inverse correlation between miR-378a-3p and IL-33 levels (*Pearson* correlation *r* = −0.67, *P* = 0.047). Furthermore, we measured the levels of the miR-378a-3p hosted gene, the PPARGC1B-mRNA, and found it decreased at 3 and 6 h after TNFα stimulus (Figure 7E) (*P* = < 0.05, *P* = < 0.001, respectively). The miR-378a-3p expression correlated directly with PPARGC1B mRNA levels (*Pearson* correlation *r* = 0.7, *P* = 0.04). Increased IL-8 mRNA levels confirmed the effect of TNFα on the cells, which was observed after 3 h (Figure 7F) (*P* = < 0.05). The mRNA levels of genes involved in microRNAs biogenesis, AGO1 and AGO2, were not affected by TNFα, suggesting that microRNAs expression changes are mainly related to inflammation (Figures 7G,H). This implies that TNFα is a regulator of miR-378a-3p expression through its host gene (PPARGC1B) in IEC.

## Discussion

In the last decade, the role of microRNAs in the intestinal homeostasis and IBD pathophysiology has been related mainly with the impairment of the epithelium barrier and inflammatory response (36). Accordingly, our data shows an altered microRNA profile expression related to the inflammatory context. In this work, we show deregulation of a set of microRNAs (miR-223-3p and miR-147b) (Table 3), whose have been founded increased in intestinal mucosa of IBD patients and were associated with macrophage polarization to an anti-inflammatory profile in a murine model (42, 43). Accordingly, a set of genes in our gene expression microarray were associated with pathways, molecular and cellular functions reflecting the UC inflammatory process. Delving into the microRNA profile, we found eight microRNAs from the miR-378 family, whose levels were decreased in inflamed vs. non-inflamed mucosa. Interestingly, these microRNAs, sharing the same target sequence, are hosted in genes that are located in different coding and non-coding genome regions, and this microRNAs redundancy might ensure effective regulation of their target genes. Hence, the down-regulation of the whole miR-378 family could represent a more efficient mechanism for IL-33 control, which signifies a possible additive effect. Moreover, each microRNA from this family is coded in different genome regions that could implicate distinctive regulation pathways for each one. This verifies the complex microRNA networking that might impact on IL-33 expression in a temporal way during inflammation. For our study, we selected three members of the miR-378 family (miR-378a-3p, miR-422a, and miR-378c) to evaluate their expression in biopsies samples. In particular, we observed decreased levels of miR-422a, and miR-378a-3p in inflamed biopsies compared to samples from inactive patients. Diminished levels of both microRNAs have been measured in gastric and colorectal tumor tissue, together with their deregulating involvement in metabolic pathways related to lipid metabolism (44–46). Thus, microRNA levels might be reflecting the metabolic shift that occurs in inflamed intestinal mucosa, described previously in ulcerative colitis, as associated mainly to microbiota deregulation with upper lipid metabolism and lower glucose uptake (47).

Our studies focused on the miR-378a-3p, whose target genes have been examined in processes of differentiation and proliferation (48, 49). Our results showed an inverse correlation of miR-378a-3p with IL-33 levels in mucosa of UC patients and healthy subjects that could evidence a mechanistic relationship between these molecules. Also, the levels of miR-378a-3p and IL-33 determined in this research confirm previous reports in UC (11, 23). At present, other examples of microRNAs regulating IL-33 include miR-487b, described as a negative regulator in murine macrophages, which decreases co-stimulatory molecule production and therefore the inflammatory response (37). Due to the cell sample complexity, IL-33 production by macrophages was not possible to observe, although this does not rule out that it can be regulated by miR-487b. Recently, another example was miR-200b and miR-200c which were described as post-transcriptional regulators of IL-33 in allergic airway inflammation disease (50), and were confirmed by our microarray data analysis showing decreased levels of both microRNAs in inflamed mucosa (−1.29 and −1.35, respectively) and we cannot dismiss their effect on IL-33 in the intestinal mucosa of UC patients. In regard to the indirect regulation of IL-33 by microRNA, miR-29a has also been described as a direct negative regulator of sST2 mRNA in a murine model of tendinopathy (51), therefore, miR-29a inhibits sST2 production affecting the activation of IL-33/ST2 axis. All these antecedents and results show the diverse microRNAs networks that might disturb IL-33/ST2 axis signaling.

**Figure 8:**
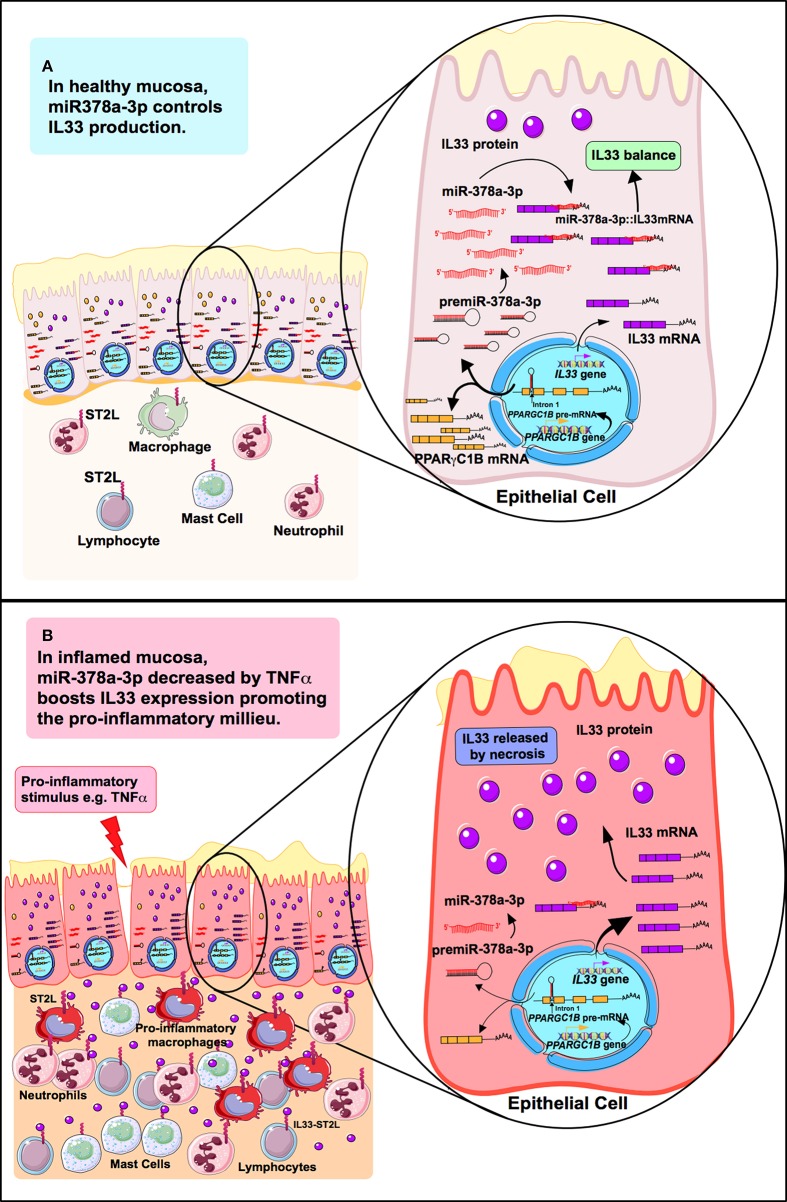
Model of IL-33 regulation by MiR-378a-3p in an inflammatory or healthy environment. **(A)** miR-378a-3p hosted in the intron 1 of PPARGC1B in the cytoplasm of epithelial cells interacts with IL-33 3′UTR mRNA controlling its production. **(B)** Inflammation condition represented by TNFα, decreases miR-378a-3p levels in epithelial cells promoting IL-33 transcription and translation, together with increased cell viability. When cells undergo necrosis, IL-33 release occurs and binds to ST2L, causing activation of lamina propria cells [neutrophils, mast cells, macrophages (pro-inflammatory phenotype) and T lymphocytes], perpetuating a pro-inflammatory environment in the intestinal mucosa.

MiR-378a-3p is induced in human primary adipocytes stimulated with TNFα or IL-6, however, there is no relating evidence between inflammatory mediators and miR-378a-3p in intestinal mucosa cells. Also, it still remains unclear in UC how IL-33 production is regulated by pro-inflammatory soluble factors such as TNFα, although it is understood that infliximab (anti-TNFα) decreases circulating IL-33 levels in UC patients (52), suggesting that its modulation involves TNFα signaling. We found increased miR-378a-3p levels in patients receiving anti-TNFα therapy, with diminished levels in IEC cells under TNFα (52). Our results suggest that modulation of miR-378a-3p by TNFα operates as an intermediary of IL-33 induction. A model of IL-33 regulation by miR-378a-3p in an inflammatory or healthy environment is represented in Figure 8. Our observation in IEC cells does not dismiss that this mechanism occurs in other IL-33 sources such as endothelial cells, where miR-378a-3p has been described as being one of the most expressed microRNAs (53) and is a potential IL-33 regulator in those cells. In addition, we found that miR-378c is reduced in mucosa of UC patients by microarrays and increased in patients under anti-TNFα therapy. MiR-378c down-regulation has been associated with diverse cancer, including colorectal and gastric (54–56), and related to a predictive role in cervical squamous cell carcinoma survival (57). As with miR-378a-3p, miR-378c might also be implicated in UC pathophysiology with TNFα related to its regulation; however, complementary studies are essential to gain insight into the biology participation of miR-378c in those diseases.

Remarkably, while miR-378a-3p is highly conserved between species (45) its target sequence in IL-33 is not (58), which is a common miRNA-target feature between distantly related animals (59). This highlights the importance of studying the interaction of miR-378a-3p and IL-33 in UC pathogenesis in human sample patients.

Our results showed high IEC cell viability when the content of miR-378a-3p is repressed and IL-33 increased. Decreased levels of miR-378a-3p have been related to colorectal cancer progression which targets cell cycle genes thus, promoting cell growth (60). Moreover, increased expression of IL-33 has been associated with cellular growth and proliferation in cancer cells (41). In inflamed intestinal mucosa, the effect of miR-378a-3p might be related to the re-epithelialization process, however, further studies are necessary to prove this hypothesis. Alternatively, the risk of colorectal cancer is elevated in UC patients and increases with disease extension and duration (61). Inflammatory molecules have been implicated in tumor microenvironment (62), and in line with our data, miR-378 family members were found decreased in stage II tumor colon cancer (63). This new knowledge emphasizes the relevance of inflammatory molecules involved in UC cancer progression; however, more studies are necessary to understand the implications of miR-378 and IL-33 in colon cancer risk for UC patients.

The miR-378a-3p is located in intron 1 of the PPARGC1B gene (PPARG coactivator 1 beta, 5q32), with levels found deregulated in intestinal mucosa of UC patients (23). We showed a direct correlation between both molecules in IEC suggesting that the microRNA might depend on the *PPARGC1B* transcriptional activity (see proposed mechanism, Figure 8). PPARGC1B protein is involved in the control of genes associated with mitogenesis and mitochondrial metabolism (64), energy production and biogenesis (65), and highly expressed in intestinal epithelium of the crypts and villus axis (66). Furthermore, *Ppargc1b* enterocyte knockout mice present a decreased expression of metabolic pathways (66). Our gene expression microarray results showed diminished levels of *PPARGC1B* (−2.1 FC) in inflamed mucosa and altered content of mRNAs associated with mitochondrial dysfunction (IPA analysis). In relation to UC pathogenesis, a decreased colonic activity of mitochondrial complex II, III, and IV from aUC patients possibly indicates impaired mitochondrial function (67). Additionally, miR-378a-3p and its host gene, have been associated with regulation of metabolic pathways as well (45). In our data, decreased levels of miR-378a-3p might reflect a metabolic shift in inflamed mucosa, possibly associated with an increment of energy expenditure and ROS overproduction in aUC patients (67). Thus, miR-378a-3p might converge a metabolic regulation with cytokine control role in the intestinal mucosa.

## Conclusion

In conclusion, this study addressed a novel mechanism of IL-33 regulation by miR-378a-3p in colonic epithelium cells under inflammatory conditions. Thus, inhibition of miR-378a-3p by inflammation enhanced IL-33 protein levels in IEC, highlighting genes and microRNAs associated with metabolic shifts in ulcerative colitis, possibly impacting on soluble inflammatory factors (Figure 8). This research contributed to an in-depth analysis of a microRNA controlling IL-33 levels in an inflammatory environment and hopefully represents the basis of new biomarkers or a therapeutic target grounded on microRNA regulation for UC.

## Data Availability Statement

The datasets generated for this study can be found in the GSE133061, https://www.ncbi.nlm.nih.gov/geo/query/acc.cgi?acc=GSE133061.

## Ethics Statement

The studies involving human participants were reviewed and approved by Sub-direction of Research, Local Ethics Committee from Clínica Las Condes, Santiago, Chile. The patients/participants provided their written informed consent to participate in this study.

## Author Contributions

KD-C performed most of the experiments and analysis, designing, and drafting of the manuscript. DD-J, MD, and GL contributed to design and achieve IL-33 detection in biopsies samples and HT-29 experiments. MO-M accomplished IPA and microarray analysis. RQ contributed to study design and sample acquisition. DS and MM enrolled UC and healthy individuals. JAC and XX performed microarray bioinformatics analysis. GG and JX designed and developed the microRNA sensor vector tools. JC developed the pri-miR lentiviral tool. RS-R supported the designing and results analysis of sensor vector experiments. M-JG prarticipated in the analysis and discussion of results. AC contributed with RNA: microRNA interaction analysis. MH contributed to study design, supervised work. All the authors contributed to the drafting and discussion of the manuscript.

### Conflict of Interest

The authors declare that the research was conducted in the absence of any commercial or financial relationships that could be construed as a potential conflict of interest.
