# Supplementary figures and images for: Inhibition of miR-378a-3p by Inflammation Enhances IL-33 Levels: A Novel Mechanism of Alarmin Modulation in Ulcerative Colitis
Source: Front Immunol. 2019 Nov 20;10:2449. doi: 10.3389/fimmu.2019.02449 (PMC6879552; doi:10.3389/fimmu.2019.02449)

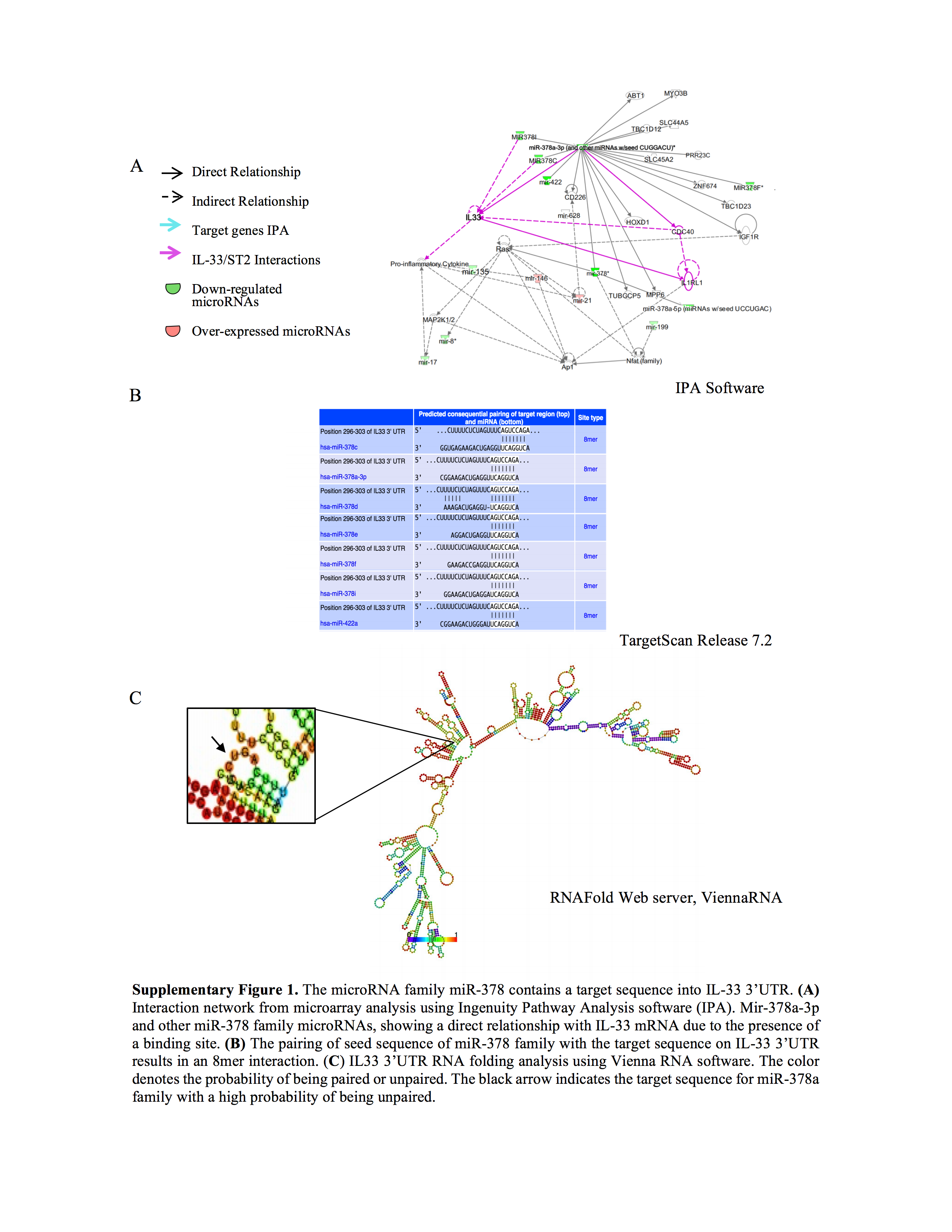

Supplement: Supplementary file 1 [file Image_1.TIFF]

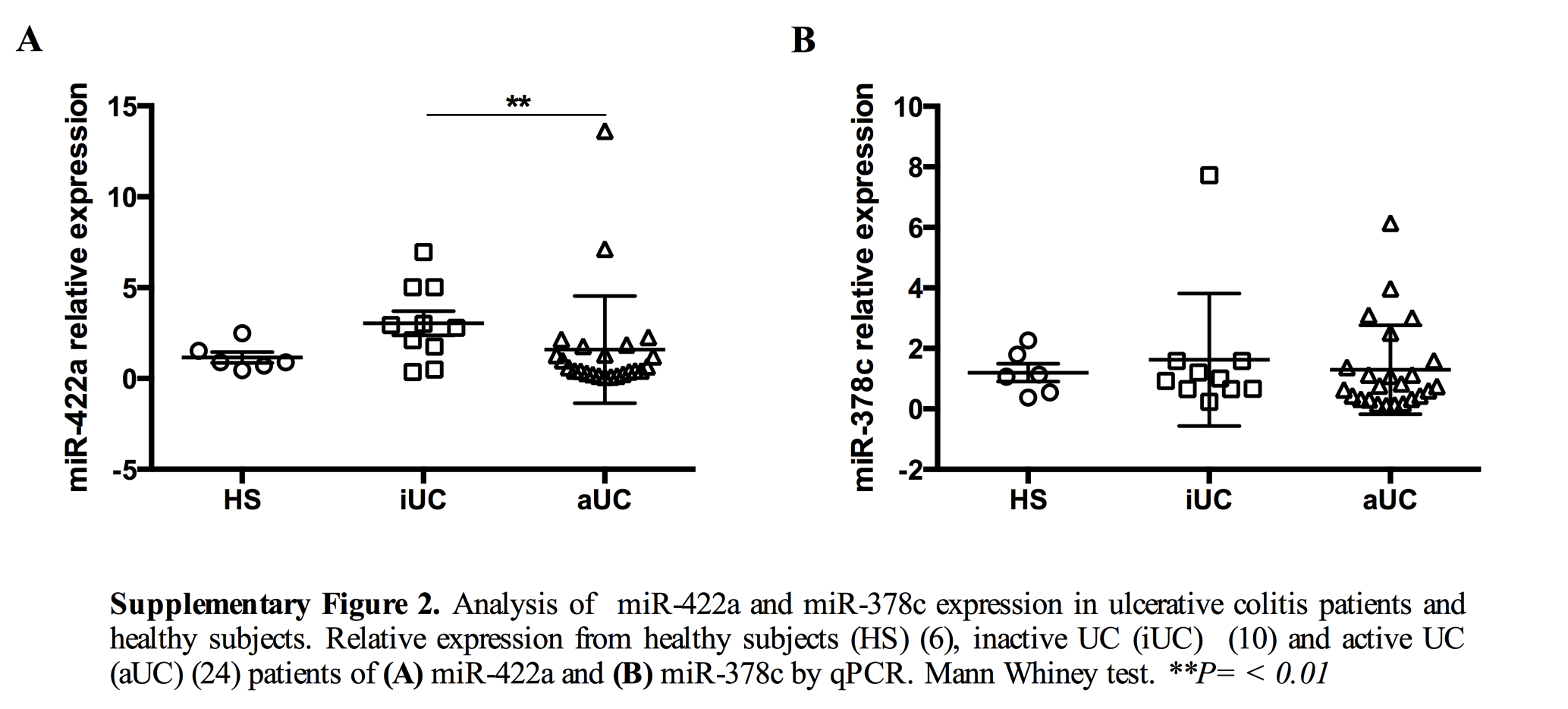

Supplement: Supplementary file 2 [file Image_2.TIFF]

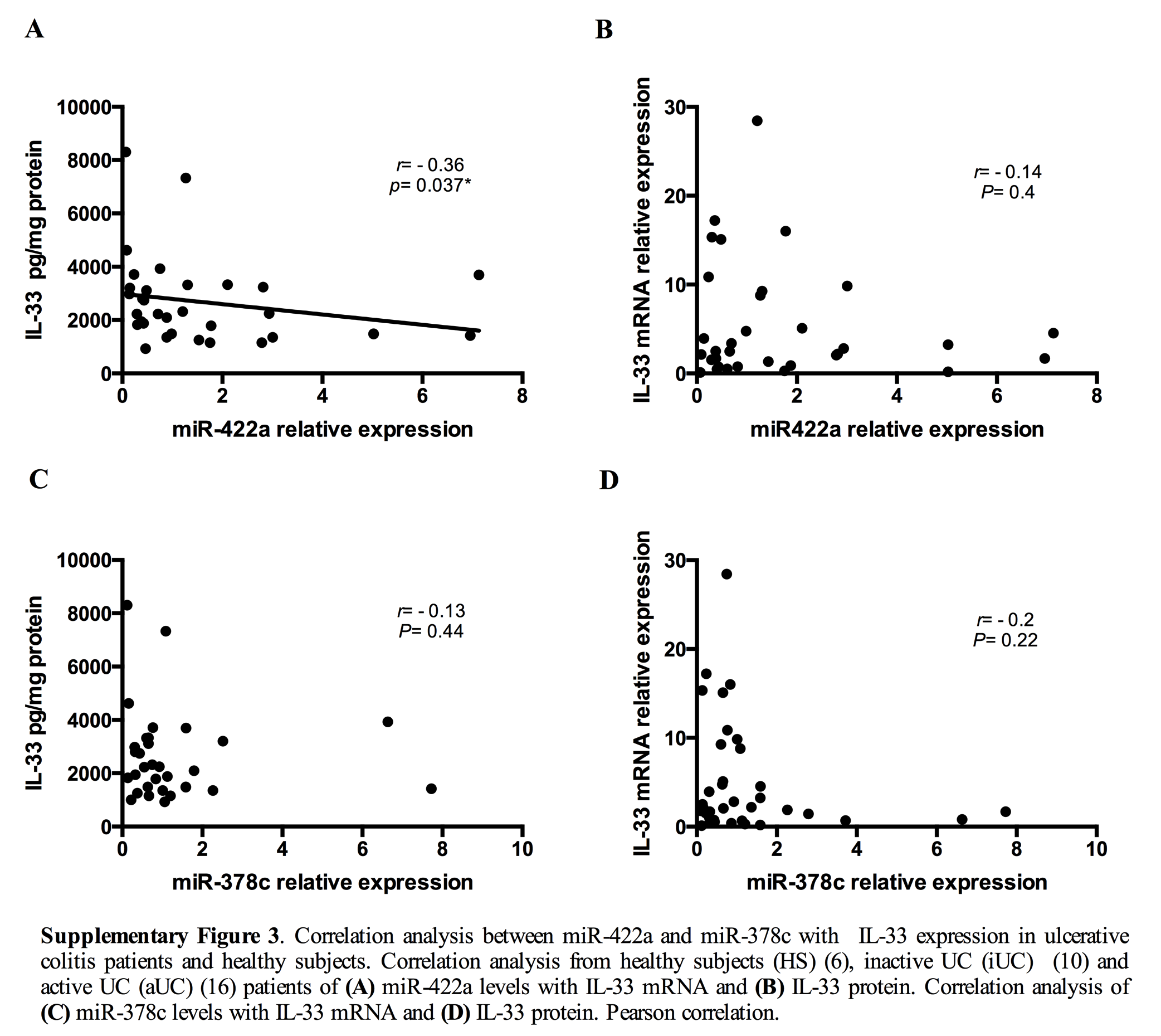

Supplement: Supplementary file 3 [file Image_3.TIFF]

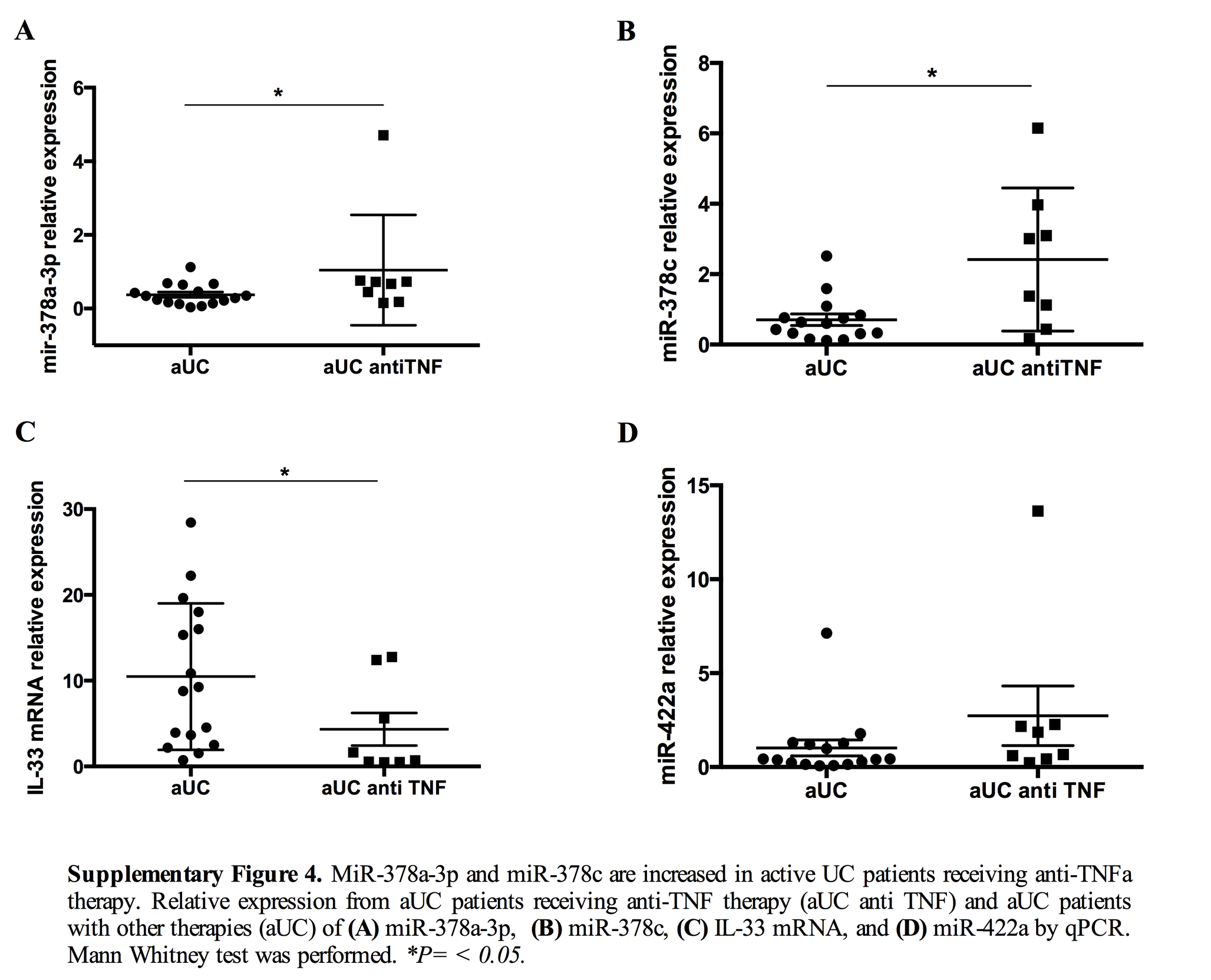

Supplement: Supplementary file 4 [file Image_4.TIFF]
